# Supplementary material for: Deletion of wfs1 Impairs Oligodendrocyte Precursor Cells Dorsal Distribution and Myelination Through the wfs1-hmgcs1 Axis in Zebrafish
Source: Biology (Basel). 2026 Aug 16;15(16):1402. doi: 10.3390/biology15161402 (PMC13510151; doi:10.3390/biology15161402)
Supplement: Supplementary file 1 [file biology-15-01402-s001.zip › File S1-Transcriptome sequencing data and differentially expressed genes (DEGs) in wfs1-- zebrafish at 5 dpf/2.KEGG/wfs1KOvsWT/DOWN/src/dre00260.html]

dre00260


- dre:550524

- Down regulated genes

550524\_1(-2.57926437647488)

- dre:394103

- Down regulated genes

394103\_1(-2.48669285078694)

- dre:64607

- Down regulated genes

64607\_1(-2.39285410667541)

- dre:541429

- Down regulated genes

541429\_1(-2.0586358059379)

- dre:550524

- Down regulated genes

550524\_1(-2.57926437647488)

- dre:541329

- Down regulated genes

541329\_1(-1.63898505916822)

Close
